# Incorporation of the novel bacterial blight resistance gene *Xa38* into the genetic background of elite rice variety Improved Samba Mahsuri

**DOI:** 10.1371/journal.pone.0198260

**Published:** 2018-05-29

**Authors:** Arra Yugander, Raman M. Sundaram, Kuldeep Singh, Duraisamy Ladhalakshmi, Lella V. Subba Rao, Maganti Sheshu Madhav, Jyothi Badri, Madamsetty Srinivas Prasad, Gouri Sankar Laha

**Affiliations:** 1 ICAR-Indian Institute of Rice Research, Hyderabad, India; 2 ICAR-National Bureau of Plant Genetic Resources, New Delhi, India; Louisiana State University, UNITED STATES

## Abstract

Bacterial blight (BB) in rice caused by *Xanthomonas oryzae* pv. *oryzae* (*Xoo*) is a major global production constraint, particularly in irrigated and rain-fed lowland areas. Improved Samba Mahsuri (ISM) is an elite, high-yielding, fine-grain type, BB-resistant rice variety possessing three BB-resistant genes (*Xa21*, *xa13* and *xa5*) and is highly popular in the southern parts of India. As the BB pathogen is highly dynamic and the evolution of pathogen virulence against the deployed resistance genes is common, we added a novel BB-resistant gene, *Xa38*, into ISM through marker-assisted backcross breeding (MABB) to increase the spectrum and durability of BB resistance. The breeding line PR 114 (Xa38) was used as the donor for *Xa38*, whereas ISM was used as the recurrent parent. Foreground selection was conducted using PCR-based gene-specific markers for the target genes, whereas background selection was conducted using a set of polymorphic SSR markers between the parents and backcrossing that continued until the third generation. Eighteen homozygous BC_3_F_2_ plants possessing all four BB-resistant genes in the homozygous state and with a recurrent parent genome (RPG) recovery of more than 92% were identified and advanced to the BC_3_F_6_ generation. These 18 backcross-derived lines (BDLs) exhibited very high level of resistance against multiple *Xoo* strains and displayed agro-morphological traits, grain qualities and yield levels similar to or better than those of the recurrent parent ISM.

## Introduction

Rice is considered essential for millions of Asians because of the immense influence on their culture, diets and economic condition and is the most important and dominant staple food crop in Asia [1]. Approximately 92% of rice is grown and consumed in Asia, which encompasses 55% of the world population [2]. To keep pace with the increasing population growth rate, estimates are that rice production must increase to approximately 136 million tonnes by 2050 [3]. One of the primary constraints in achieving this target is the increased incidence of several pests and diseases. Intensive cultivation practices involving the widespread cultivation of a few high-yielding rice varieties with a narrow genetic base coupled with heavy dependency on chemical fertilizers and pesticides and apparent changes in the climate have resulted in increased incidence of many diseases, including bacterial blight (BB) in rice [4].

BB caused by *Xanthomonas oryzae* pv. *oryzae* (*Xoo*) remains a primary production constraint in rice in India and in most of east and southeast Asian countries [5]. This disease primarily occurs in epidemic proportions in monsoon (wet) season, particularly in irrigated and rain-fed lowland ecosystems [6]. Analyses of disease survey data from the past 34 years in several rice growing regions of India indicate that the disease has increased in both intensity and geographical distribution, as exemplified by several reports of BB occurrence in recent years in epidemic form [4] and of the extent of yield loss due to this disease, which can be as high as 50% or more [7].

Chemical control against this disease has not been very successful in spite of extensive evaluation of several chemicals and antibiotics [6]. Therefore, major emphasis is placed on the development and deployment of BB-resistant rice varieties [8, 9]. To date, more than 40 BB-resistant genes are identified from various sources [10–14]. However, the pathogen is highly diverse in nature, particularly in India [5], and several studies indicate that cultivars with single BB-resistant genes do not provide broad-spectrum resistance [5, 15–17]. Therefore, pyramiding of multiple BB-resistant genes into rice varieties through marker-assisted backcross breeding (MABB) is the most effective approach to develop durable BB resistance [18–26].

Joseph et al. [27] pyramided two major BB-resistant genes, *xa13* and *Xa21*, into the genetic background of the highly popular basmati rice variety Pusa Basmati 1, which was released for commercial cultivation as ‘Improved Pusa Basmati 1’ during 2007 [28]. However, some *Xoo* strains are virulent on varieties possessing a combination of *Xa21* and *xa13* [5, 14, 16]. Sundaram et al. [20] pyramided three BB-resistant genes, *Xa21*, *xa13* and *xa5*, into the genetic background of the elite mega-variety of rice, Samba Mahsuri, which is a highly popular medium-slender and fine-grain type rice variety. The improved, BB-resistant version of Samba Mahsuri, named ‘Improved Samba Mahsuri’ (ISM), possesses the high yield and elite grain quality attributes of Samba Mahsuri and a high level of resistance against BB disease. Therefore, ISM was released for commercial cultivation in south India and parts of eastern India in 2008 [28]. ISM presently occupies an area of more than 80,000 hectares [4] and has shown a good level of resistance in the fields of farmers [25]. However, the variety has shown marginally longer lesions (disease score of 5 based on Standard Evaluation System for Rice) [29] upon artificial inoculation at a few BB hot spot locations in the All India Coordinated Plant Pathology trials [30]. Therefore, the introduction of additional resistance gene(s) into ISM to increase the durability and spectrum of resistance of this highly popular variety is desirable. Earlier, Cheema et al. [31] identified the novel, broad-spectrum BB-resistant gene *Xa38* from *Oryza nivara*, and the gene was mapped on the long arm of chromosome 4. The gene was further fine-mapped to a 38.4 kb region and has provided a high level of resistance against multiple virulent isolates of the BB pathogen [10]. The gene was recently introgressed into an elite basmati rice variety, PB 1121, and the improved lines exhibited a high level of BB resistance [14]. In the present study, we used the MABB strategy to introgress *Xa38*, a major BB-resistant gene, into the ISM background with the objective to increase the durability and spectrum of resistance of this highly preferred rice variety.

## Materials and methods

### Plant materials

The breeding line PR 114 (Xa38), which is an introgressed line derived from a cross between PR114 and *Oryza nivara* S. D. Sharma & Shastry possessing the novel BB-resistant gene, *Xa38* [31], was used as the donor parent. Improved Samba Mahsuri (ISM) possessing three major BB-resistant genes, *Xa21*, *xa13* and *xa5*, was used as the recurrent parent. Earlier, our research group developed ISM by pyramiding three BB-resistant genes in the genetic background of Samba Mahsuri through MABB [20]. The rice genotypes Samba Mahsuri, Taichung Native 1 (TN1) and IRBB21 (possessing *Xa21*) were used as checks in disease screening experiments.

### Crossing scheme and MABB for introgression of *Xa38* in ISM

**Fig 1:**
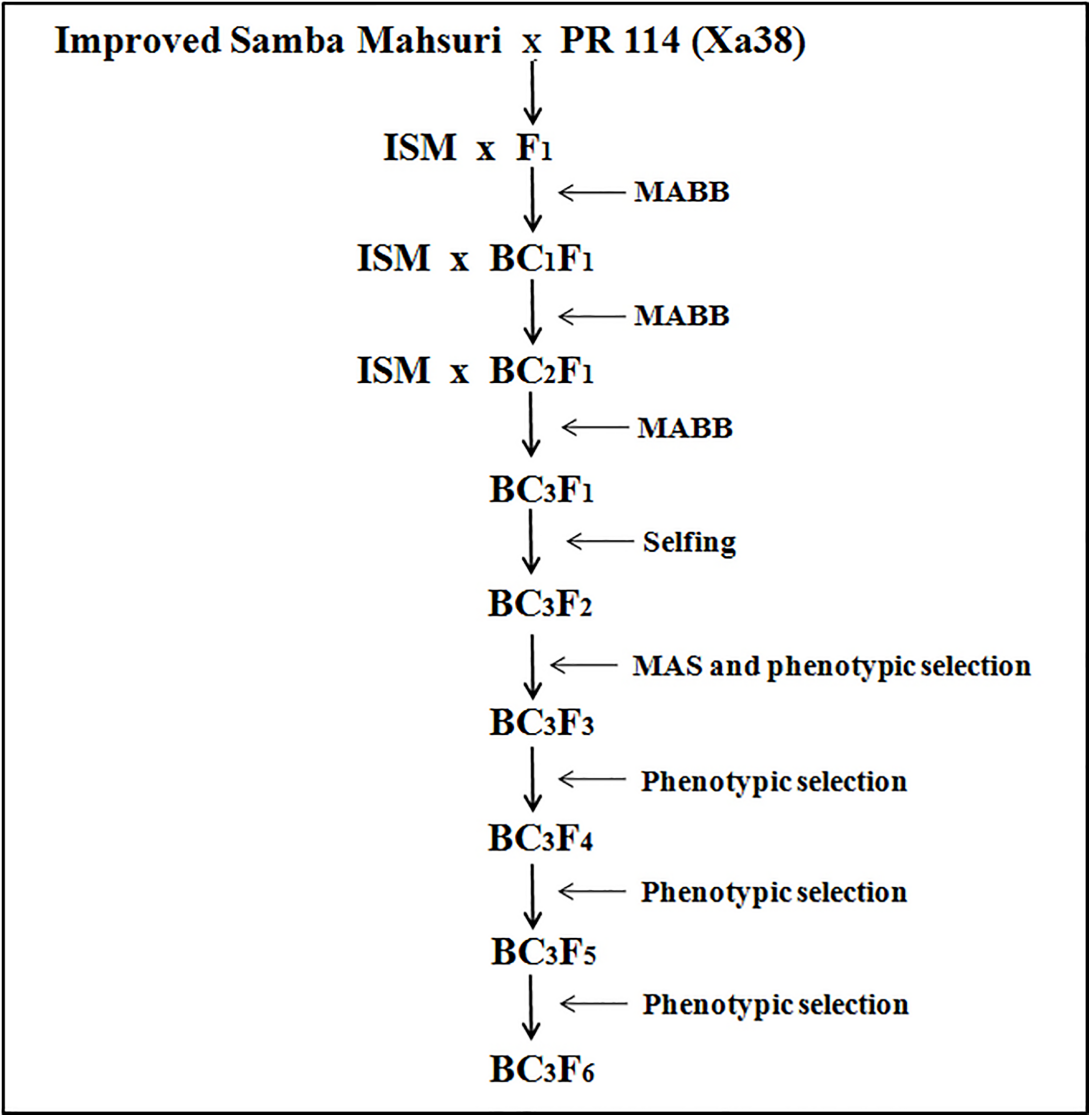
Crossing scheme adopted for the marker-assisted introgression of *Xa38* in the genetic background of Improved Samba Mahsuri (ISM).

Introgresssion of BB-resistant gene *Xa38* into the genetic background of ISM was performed by crossing between ISM and PR 114 (Xa38) during 2012. True F_1_ plants were identified using the gene-specific marker for *Xa38* (Os04g53050-1) [10]. The true F_1_s were then backcrossed with the recurrent parent, ISM, to generate BC_1_F_1_s_,_ which were subjected to foreground selection using the *Xa38* gene-specific marker and background selection using a set of parental polymorphic SSR markers. Background selection is a good approach to screen the lines having higher recurrent parent genome (RPG). A single positive BC_1_F_1_ plant with the maximum RPG recovery was then backcrossed with ISM to generate BC_2_F_1_s_,_ and the MABB process was continued up to the BC_3_ generation (Fig 1). In the BC_3_F_1_ generation, plants that had BB-resistant genes *Xa21*, *xa13* and *xa5* in the homozygous condition and *Xa38* in the heterozygous condition and possessed maximum RPG recovery were identified and selfed to generate BC_3_F_2_s. Among the BC_3_F_2_ plants, the homozygous plants for all four genes were identified through marker-assisted selection and then subjected to background selection for identification of plants that had the maximum recovery of the RPG. Such BC_3_F_2_ plants that possessed all four genes in the homozygous state with maximum RPG recovery and that were phenotypically similar or superior to ISM were identified and advanced to the BC_3_F_6_ stage for further evaluation.

For detection of the target genes and background selection, genomic DNA was isolated from parents and backcross-derived lines (BDLs) by following the protocol described in Zheng et al. [32]. Detection of the desired genes, *Xa38*, *Xa21*, *xa13* and *xa5*, in the parents and backcross-derived lines were conducted through PCR and agarose gel electrophoresis using gene-specific markers, viz., Os04g53050-1 for *Xa38* [10], pTA248 for *Xa21* [33], xa13-Prom for *xa13* [34] and 10603-T10Dw for *xa5* [35]. For background selection, SSR loci polymorphic between the donor [PR 114 (Xa38)] and the recurrent parent (ISM) were identified by screening 366 rice SSR markers spread across the 12 chromosomes of rice as per the procedure described by Sundaram et al. [20]. A total of 83 parental polymorphic SSR markers, which were fairly well distributed throughout the 12 chromosomes of rice (i.e., ~5–7 polymorphic markers per chromosome), were identified and used to genotype foreground-selected plants at each backcross generation to identify a single plant with the maximum RPG recovery. More polymorphic SSR markers were used on the target chromosome (i.e., 18 SSRs on chr. 4 on which *Xa38* is located) to reduce the linkage drag in the genomic region around the target resistance gene. Using the polymorphic marker data, a schematic map depicting the genomic contribution of donor and recurrent parents was prepared using Graphical Genotype (GGT) Version 2.0 software [36] to identify BDLs (BC_3_F_2_) possessing maximum recovery of RPG.

### Screening for BB resistance

A total of 8 virulent *Xoo* strains were used in the present study: IX-015 (Aduthurai, Tamil Nadu), IX-020 (Hyderabad, Telangana), IX-116 (Panvel, Maharashtra), IX-133 (Raipur, Chhattishgarh), IX-200 (Pantnagar, Uttarakhand), IX-220 (Kaul, Haryana), IX-221 (Karnal, Haryana) and IX-279 (Tanuku, Andhra Pradesh). These strains were selected from our core collection and belonged to different pathotypes [5]. The parents and selected four-gene pyramid BDLs (possessing *Xa38*, *Xa21*, *xa13* and *xa5*) from the BC_3_F_6_ generation were evaluated for their reaction to BB under glasshouse conditions during the wet season of 2016 following the clip-inoculation method [37] as described earlier [5]. For each genotype/*Xoo* strain combination, 12–15 fully expanded leaves were clip inoculated. Lesion lengths were measured 15 days after inoculation. Three observations were recorded for each genotype, and then the average lesion length was calculated. Average lesion length up to 3 cm was scored as resistant (R), 3–6 cm as moderately resistant (MR), 6–9 cm as moderately susceptible (MS) and more than 9 cm as susceptible (S) [15].

### Evaluation for agro-morphological characteristics

Twenty-five-day-old seedlings of selected BDLs and parents were transplanted into mini-plots (2 x 3 m^2^) in the experimental field of ICAR-Indian Institute of Rice Research, Hyderabad, India, with a spacing of 15 x 20 cm in three replications during the wet season in 2016. Data were recorded for different agro-morphological traits: plant height (cm), number of panicles/hill, days to 50% flowering, number of grains/panicle, panicle length (cm), 1000 grain weight (g), grain weight/plant and grain type, as explained in Hari et al. [38]. The experiment was conducted following Randomized Complete Block Design (RCBD) and the data were analyzed statistically following the protocol described in Gomez and Gomez [39].

### Evaluation of the grain quality characters

Backcross-derived improved lines of ISM and the parents (3 months after harvest at 12–14% moisture content) were evaluated for different physicochemical characters: hulling (%), milling (%), head rice recovery (%), L/B ratio, kernel length after cooking (KLAC), elongation ratio, alkali spreading value and amylose content (%) of the grains; standard procedures were followed [20, 29, 40–42].

## Results

### Marker-assisted introgression of *Xa38* into ISM

Of 70 F_1_ plants screened, 67 plants were positive for *Xa38* (*Xa38xa38*, i.e., true heterozygotes). At BC_1_F_1_, 87 of 160 plants were positive for *Xa38*, and the recovery of RPG among the positive BC_1_F_1_ plants ranged from 74.7% to 77.1%. Furthermore, these plants showed a very high level of BB resistance when inoculated with a virulent strain of *Xoo* (IX-220). A single true BC_1_F_1_ plant (#GLY-1-19-34) with the maximum RPG recovery (77.1%) was backcrossed to ISM to generate BC_2_F_1_s (180 seeds). Of 80 BC_2_F_1_ plants raised and screened, 45 were positive for *Xa38* when screened with the *Xa38* gene-specific marker, and the percentage of RPG recovery in these plants ranged from 86.7% to 90.3%. Furthermore, all showed a high level of resistance against BB (with lesion lengths less than 0.5 cm) when inoculated with a virulent *Xoo* strain, IX-220. A single selected positive BC_2_F_1_ plant (#GLY-1-19-34-61) with the maximum RPG (90.3%) was then backcrossed with ISM to generate BC_3_F_1_s_._ Of 74 BC_3_F_1_ plants raised and screened, 40 were positive for *Xa38*, and the RPG recovery in these positive BC_3_F_1_ plants ranged between 92.7% and 95.1%.

**Fig 2:**
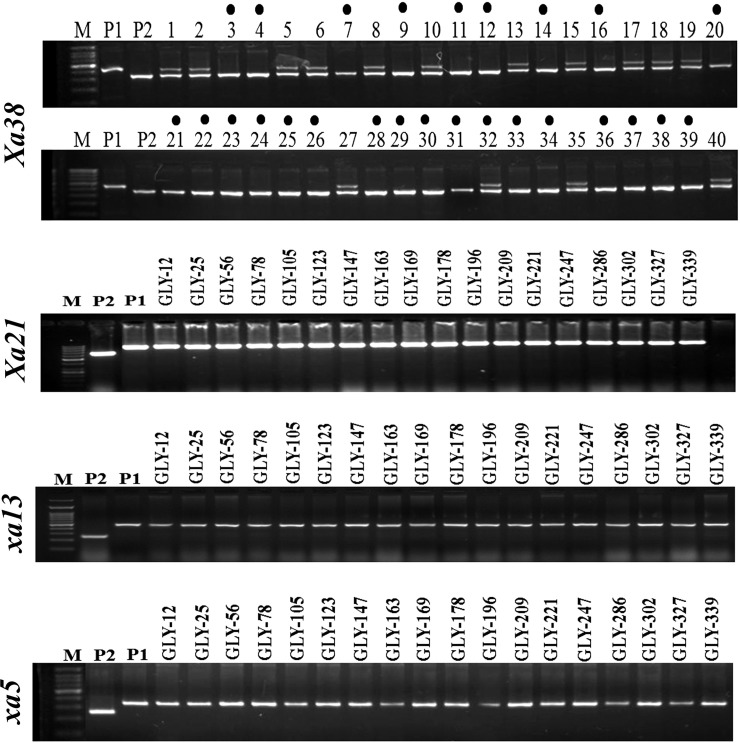
Molecular detection of *Xa38*, *Xa21*, *xa13* and *xa5* in the BC_3_F_2_ plants through PCR using gene-linked markers Os04g53050-1, pTA248, xa13-Prom and 10603-T10Dw, respectively. The marks shown on the top of the gel represent the positive BC_3_F_2_ plants for *Xa38*. Lane M: 100 bp molecular weight ladder; P1-Recurrent parent, Improved Samba Mahsuri; P2-Donor parent, PR 114 (Xa38).

**Fig 3:**
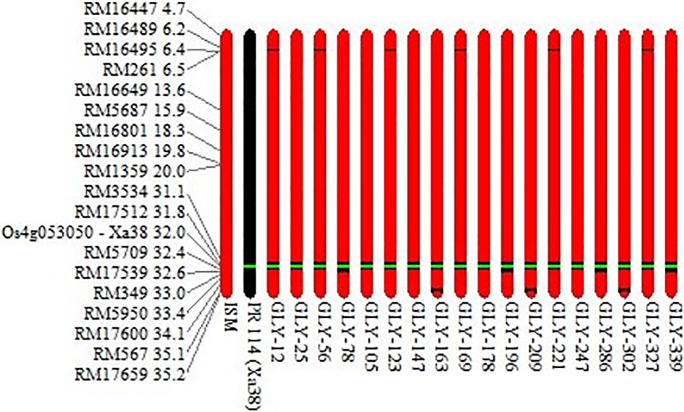
Analysis of genome introgression associated with the BB-resistant gene *Xa38* on chromosome 4 in the eighteen selected BDLs of Improved Samba Mahsuri. A genomic region limited to ∼0.6 Mb has been only introgressed from the donor parent PR 114 (Xa38) in the selected BDLs. The position of each polymorphic SSR marker in Mb on Chr. 4 is given adjacent to each marker.

A single selected BC_3_F_1_ (#GLY-1-19-34-61-17) plant with the maximum RPG recovery and very high level of BB resistance was selfed to generate BC_3_F_2_s. Of 350 BC_3_F_2_ plants analyzed, 91 were homozygous positive for all four BB-resistant genes, viz., *Xa38*, *Xa21*, *xa13* and *xa5* (Fig 2). Of these plants, 18 plants, which were phenotypically similar or superior to ISM, were advanced up to the BC_3_F_6_ generation by adopting the pedigree method of selection. When the selected BC_3_F_6_ lines were analyzed for linkage drag near the target resistance gene *Xa38*, most possessed an introgression of ≤ 0.6 Mb segment (0.2 Mb at the proximal end and 0.4 Mb at the distal end) with respect to *Xa38* of the donor parent genomes (Fig 3).

### Evaluation of BB resistance of parents and BDLs

**Fig 4:**
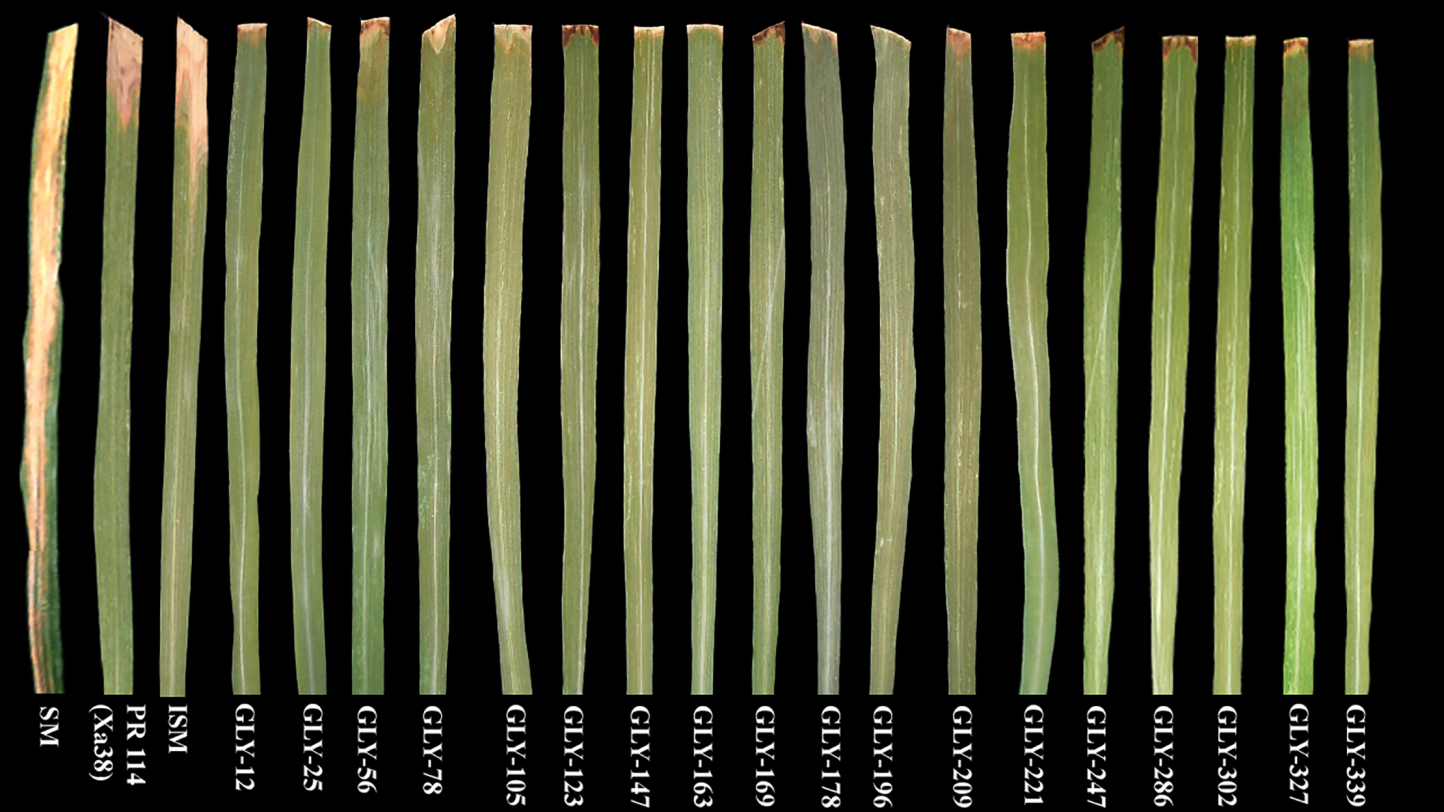
Reaction of the selected BDLs at the BC_3_F_6_ generation against bacterial blight of rice. Forty-day-old plants were clip inoculated using a 3-day-old culture of IX-220 *Xoo* strain, and the lesion lengths (cm) were measured after 15 days of inoculation.

Eighteen selected BC_3_F_6_ lines were evaluated for their resistance against BB using eight virulent *Xoo* strains under glasshouse conditions during the wet season of 2016. The details of the BB reactions of the BDLs and the parents are presented in Table 1. The recurrent parent ISM, which possessed three BB-resistant genes (*Xa21*, *xa13* and *xa5*), showed a lesion length of 2.07–6.43 cm. The donor parent PR 114 (Xa38), which possessed the novel BB-resistant gene *Xa38*, showed a lesion length of 1.17–9.80 cm. The checks, Samba Mahsuri and TN1, showed highly susceptible reaction with lesion length ranging from 18.07 to 28.97 cm. IRBB21, possessing BB-resistant gene *Xa21*, showed a moderate to susceptible reaction with lesion length ranging from 5.8 to 9.8 cm. All the BDLs, which possessed all four BB-resistant genes, showed very high level of resistance to BB with lesion length less than 0.5 cm (Fig 4; Table 1). The data indicated that the addition of BB-resistant gene *Xa38*, imparted an increased level of resistance as demonstrated by the reduced lesion length.

**Table 1:** Phenotypic reaction of backcross-derived lines, parents and checks against *Xoo* strains.

Code	IX-015	IX-020	IX-116	IX-133	IX-200	IX-220	IX-221	IX-279
GLY-12	0.33 ± 0.09	0.33 ± 0.09	0.47 ± 0.03	0.30 ± 0.06	0.43 ± 0.07	0.37 ± 0.07	0.47 ± 0.03	0.50 ± 0.06
GLY-25	0.43 ± 0.09	0.30 ± 0.09	0.37 ± 0.03	0.37 ± 0.07	0.37 ± 0.09	0.43 ± 0.03	0.40 ± 0.06	0.33 ± 0.03
GLY-56	0.30 ± 0.09	0.23 ± 0.03	0.50 ± 0.06	0.33 ± 0.07	0.50 ± 0.06	0.50 ± 0.06	0.33 ± 0.03	0.43 ± 0.09
GLY-78	0.37 ± 0.09	0.37 ± 0.07	0.33 ± 0.03	0.40 ± 0.06	0.40 ± 0.06	0.33 ± 0.03	0.27 ± 0.07	0.23 ± 0.03
GLY-105	0.27 ± 0.07	0.37 ± 0.03	0.33 ± 0.03	0.27 ± 0.03	0.40 ± 0.06	0.40 ± 0.06	0.47 ± 0.07	0.30 ± 0.06
GLY-123	0.33 ± 0.03	0.30 ± 0.06	0.40 ± 0.06	0.30 ± 0.06	0.43 ± 0.07	0.33 ± 0.09	0.40 ± 0.06	0.33 ± 0.09
GLY-147	0.30 ± 0.06	0.43 ± 0.03	0.43 ± 0.03	0.37 ± 0.09	0.43 ± 0.07	0.40 ± 0.06	0.33 ± 0.09	0.30 ± 0.06
GLY-163	0.33 ± 0.07	0.33 ± 0.03	0.33 ± 0.03	0.30 ± 0.06	0.33 ± 0.03	0.33 ± 0.03	0.30 ± 0.06	0.33 ± 0.03
GLY-169	0.30 ± 0.06	0.40 ± 0.06	0.33 ± 0.09	0.30 ± 0.06	0.40 ± 0.07	0.43 ± 0.07	0.33 ± 0.03	0.27 ± 0.03
GLY-178	0.30 ± 0.09	0.33 ± 0.09	0.33 ± 0.03	0.27 ± 0.03	0.33 ± 0.03	0.33 ± 0.03	0.30 ± 0.06	0.33 ± 0.09
GLY-196	0.27 ± 0.07	0.33 ± 0.03	0.30 ± 0.06	0.30 ± 0.06	0.47 ± 0.03	0.40 ± 0.06	0.33 ± 0.03	0.37 ± 0.09
GLY-209	0.23 ± 0.03	0.27 ± 0.07	0.33 ± 0.07	0.33 ± 0.03	0.37 ± 0.03	0.33 ± 0.07	0.37 ± 0.09	0.40 ± 0.06
GLY-221	0.40 ± 0.06	0.33 ± 0.03	0.30 ± 0.06	0.30 ± 0.06	0.43 ± 0.03	0.37 ± 0.03	0.30 ± 0.06	0.33 ± 0.09
GLY-247	0.30 ± 0.06	0.23 ± 0.03	0.37 ± 0.07	0.37 ± 0.09	0.40 ± 0.07	0.40 ± 0.07	0.33 ± 0.09	0.27 ± 0.07
GLY-286	0.33 ± 0.09	0.40 ± 0.06	0.27 ± 0.03	0.27 ± 0.03	0.47 ± 0.03	0.37 ± 0.07	0.27 ± 0.07	0.33 ± 0.09
GLY-302	0.30 ± 0.06	0.33 ± 0.09	0.33 ± 0.09	0.30 ± 0.06	0.43 ± 0.03	0.43 ± 0.03	0.30 ± 0.06	0.30 ± 0.09
GLY-327	0.30 ± 0.06	0.33 ± 0.03	0.23 ± 0.03	0.33 ± 0.09	0.47 ± 0.03	0.43 ± 0.03	0.23 ± 0.03	0.23 ± 0.03
GLY-339	0.33 ± 0.06	0.23 ± 0.03	0.40 ± 0.06	0.30 ± 0.06	0.43 ± 0.03	0.40 ± 0.06	0.37 ± 0.07	0.33 ± 0.03
ISM	2.07 ± 0.28	2.83 ± 0.19	3.33 ± 0.23	2.97 ± 0.28	5.43 ± 0.29	6.43 ± 0.015	3.00 ± 0.15	3.13 ± 0.15
PR 114 (Xa38)	1.17 ± 0.20	1.43 ± 0.19	9.80 ± 0.29	3.20 ± 0.40	3.90 ± 0.29	4.13 ± 0.20	2.20 ± 0.40	3.67 ± 0.19
IRBB21	6.20 ± 0.26	7.00 ± 0.78	5.80 ± 0.29	8.73 ± 0.41	9.80 ± 0.78	6.20 ± 0.21	6.07 ± 0.87	7.07 ± 0.50
Samba Mahsuri	18.07 ± 0.92	20.8 ± 0.17	22.73 ± 0.58	18.83 ± 0.52	24.87 ± 0.46	20.87 ± 0.50	23.63 ± 0.79	24.40 ± 0.85
TN1	18.50 ± 0.75	21.43 ± 1.59	24.50 ± 0.46	19.50 ± 0.68	28.50 ± 0.56	21.17 ± 0.23	28.97 ± 0.94	25.83 ± 0.54
CV (%)	21.20	24.78	10.55	15.82	12.26	8.50	18.66	13.86
LSD (*P = 0*.*05*)	0.78	1.05	0.54	0.66	0.70	0.40	0.93	0.69

### Yield and agronomic performance of BDLs

**Fig 5:**
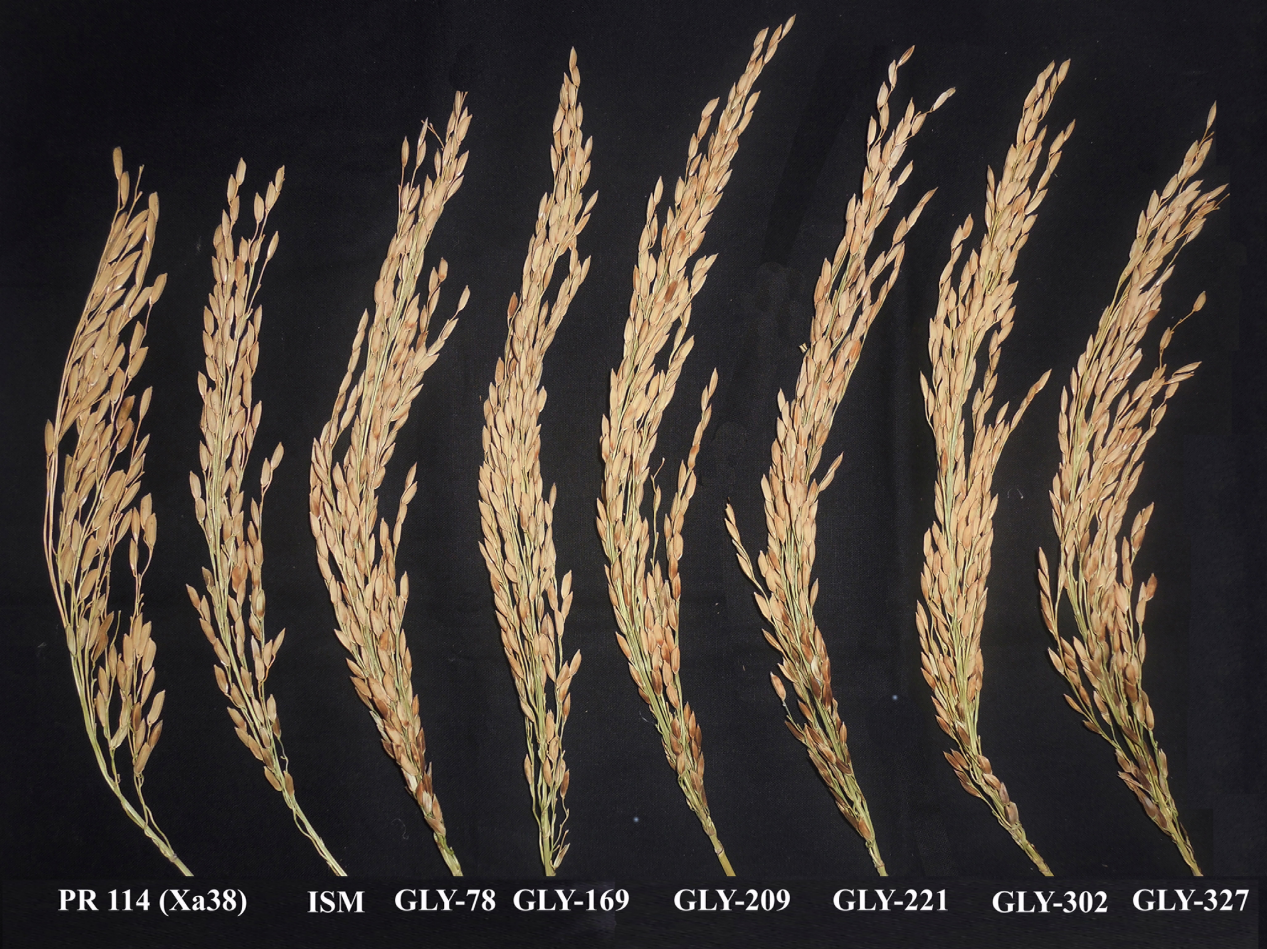
Panicles of selected BDLs at the BC_3_F_6_ generation. The backcross-derived lines GLY # 78, 169, 209, 221, 302 and 327 possessed compact panicles with grain type similar to that of Improved Samba Mahsuri.

The selected BDLs of ISM at the BC_3_F_6_ generation and their parents were evaluated for selected agro-morphological characters during the wet season of 2016. Compared with those of the recurrent parent, statistically significant differences were observed in BDLs for plant height, days to 50% flowering, number of grains/panicle and grain weight/panicle. The mean plant height of the BDLs ranged from 71.7 to 94 cm compared with 77.3 cm of the recurrent parent ISM. A few of the lines, viz., GLY-25, GLY-56, GLY-105, GLY-123, GLY-163 and GLY-209, showed significantly increased mean plant height (86–94 cm) compared with that of ISM (Table 2). Large variation occurred in days to 50% flowering (99.7–108.3 days) among the BDLs, compared with that of the recurrent parent ISM (104 days). A few of the lines, viz., GLY-105, GLY-123, GLY-147 and GLY-163, were significantly earlier by 4–5 days, whereas GLY-78 was later by 4 days than the recurrent parent ISM. Average number of grains/panicle among the BDLs ranged from 131.3 (GLY-221 and GLY-339) to 147 (GLY-209) and was significantly higher than that of the recurrent parent ISM (128 grains/panicle) (Table 2; Fig 5). The entries such as GLY #- 56, 78, 105, 123, 169, 178, 196 and 209 were highly promising with 141–147 grains/panicle. Average grain weight per plant among the BDLs was significantly higher (19.1–25.57 g) than that of the recurrent parent (18.9 g). The lines GLY-169, GLY-178 and GLY-209 were highly promising with average grain weight per plant ranging from 23 to 25 g. Although the average panicle length did not differ drastically, a few of the BDLs, such as GLY #- 25, 56, 105, 123, 178, 221, 286 and 339, were promising, with slightly larger panicles than that of the recurrent parent ISM. No significant difference was observed for the number of panicles/hill and 1000 grain weight between the BDLs and the recurrent parent. All the BDLs had a medium-slender grain type, as did the recurrent parent ISM.

**Table 2:** Agro-morphological characters of backcross-derived lines of Improved Samba Mahsuri.

Line No.	Plant height (cm)	No. of panicles/hills	Days to 50% flowering	No. of grains /panicle	Panicle length (cm)	1000 grain weight (g)	Grain weight (g)/plant	Grain type	Genome recovery (%)
GLY-12	83.67 ± 2.03	13.33 ± 0.88	105.67 ± 0.88	138.67 ± 2.73	22.50 ± 0.21	11.53 ± 0.30	21.07 ± 0.15	MS	93.98
GLY-25	94.00 ± 1.00	13.00 ± 0.58	102.67 ± 0.88	137.33 ± 1.76	23.57 ± 0.30	11.41 ± 0.08	20.20 ± 0.06	MS	95.18
GLY-56	86.00 ± 2.00	13.33 ± 0.88	101.33 ± 0.88	141.67 ± 1.76	24.77 ± 0.35	11.47 ± 0.16	21.07 ± 0.09	MS	93.98
GLY-78	81.00 ± 5.00	14.00 ± 0.58	108.33 ± 0.88	143.67 ± 1.45	21.77 ± 0.17	11.46 ± 0.13	22.97 ± 0.12	MS	93.98
GLY-105	91.00 ± 3.61	13.33 ± 1.15	100.67 ± 0.88	146.00 ± 2.31	23.00 ± 0.60	11.25 ± 0.14	21.17 ± 0.09	MS	95.18
GLY-123	93.00 ± 1.53	13.33 ± 0.88	99.67 ± 0.88	143.00 ± 3.51	23.43 ± 0.34	11.31 ± 0.32	21.07 ± 0.09	MS	93.98
GLY-147	81.00 ± 2.08	13.67 ± 0.33	100.33 ± 1.20	136.33 ± 2.91	22.70 ± 0.25	11.07 ± 0.19	19.63 ± 0.03	MS	95.18
GLY-163	77.33 ± 2.73	13.67 ± 0.67	99.67 ± 0.88	137.00 ± 2.65	21.33 ± 0.27	11.39 ± 0.12	21.17 ± 0.09	MS	92.77
GLY-169	71.67 ± 0.88	14.67 ± 0.33	101.00 ± 0.58	146.67 ± 0.88	22.77 ± 0.38	11.44 ± 0.17	23.23 ± 0.09	MS	93.98
GLY-178	73.67 ± 1.86	14.67 ± 0.88	102.67 ± 0.88	143.33 ± 1.86	23.93 ± 0.22	11.25 ± 0.10	23.47 ± 0.07	MS	95.18
GLY-196	82.33 ± 2.03	14.00 ± 0.58	104.00 ± 0.58	143.67 ± 2.19	22.67 ± 0.35	11.17 ± 0.16	22.40 ± 0.06	MS	93.98
GLY-209	78.33 ± 4.10	14.67 ± 1.20	103.00 ± 0.58	147.00 ± 2.31	22.83 ± 0.55	11.86 ± 0.14	25.57 ± 0.09	MS	92.77
GLY-221	80.00 ± 1.53	13.33 ± 0.88	102.00 ± 1.15	131.33 ± 2.40	23.27 ± 0.62	11.66 ± 0.12	19.93 ± 0.09	MS	93.98
GLY-247	81.33 ± 0.67	13.33 ± 0.33	104.67 ± 0.88	131.67 ± 1.76	21.97 ± 0.58	11.49 ± 0.36	19.73 ± 0.12	MS	95.18
GLY-286	83.33 ± 1.67	12.67 ± 1.33	104.67 ± 0.33	133.00 ± 1.15	23.33 ± 0.09	11.37 ± 0.25	19.63 ± 0.09	MS	93.98
GLY-302	89.00 ± 1.53	13.00 ± 0.00	104.33 ± 0.88	135.67 ± 0.88	22.90 ± 0.06	11.24 ± 0.34	19.13 ± 0.03	MS	92.77
GLY-327	91.33 ± 3.33	13.00 ± 0.58	103.67 ± 0.88	134.67 ± 2.33	20.93 ± 0.09	11.14 ± 0.16	19.70 ± 0.06	MS	93.98
GLY-339	80.67 ± 1.67	12.67 ± 0.33	105.00 ± 0.58	131.33 ± 2.85	23.33 ± 0.09	11.43 ± 0.21	19.10 ± 0.06	MS	93.98
ISM	77.33 ± 0.88	12.67 ± 0.88	104.00 ± 1.15	128.00 ± 1.15	21.67 ± 0.15	11.36 ± 0.18	18.97 ± 0.12	MS	
PR 114 (Xa38)	95.67 ± 0.88	11.00 ± 0.58	115.33 ± 0.88	117.33 ± 1.45	22.83 ± 0.23	14.55 ± 0.33	17.50 ± 0.06	LS	
CV (%)	4.79	9.69	1.35	2.57	3.03	1.80	0.72		
LSD (*P = 0*.*05*)	6.61	2.14	2.31	5.82	1.14	0.40	0.24		

### Evaluation of grain quality characters of the BDLs

The donor PR 114 (Xa38), which has the novel BB-resistant gene *Xa38*, has long-slender grain compared with that of ISM, with highly desirable, medium-slender grain. All the BDLs possessed medium-slender grain type and showed similarity to ISM in all the grain quality attributes analyzed, viz., hulling percentage (75–78.1% in BDLs), milling percentage (64.8–69.7%), kernel length, kernel breadth, L/B ratio, grain chalkiness, kernel length after cooking (8.4–10.5 mm), elongation ratio, alkali spreading value and intermediate amylase content (Table 3).

**Table 3:** Grain quality characteristics of parents and backcross breeding lines of ISM.

Plant material	Hulling (%)	Milling (%)	HRR (%)	KL (mm)	KB (mm)	L/B Ratio	KLAC (mm)	ER (mm)	ASV	AC (%)
GLY-12	77.60 ± 0.21	65.97 ± 0.15	61.40 ± 0.32	4.89 ± 0.07	1.78 ± 0.02	2.73 ± 0.02	8.93 ± 0.26	1.87 ± 0.03	5.0	22.41 ± 0.31
GLY-25	76.17 ± 0.38	65.80 ± 0.26	59.50 ± 0.61	4.83 ± 0.05	1.87 ± 0.01	2.60 ± 0.04	9.23 ± 0.26	1.97 ± 0.04	5.0	22.76 ± 0.55
GLY-56	77.33 ± 0.15	69.40 ± 0.21	66.73 ± 0.43	5.11 ± 0.03	1.82 ± 0.02	2.76 ± 0.03	8.87 ± 0.38	1.78 ± 0.03	6.0	23.32 ± 0.42
GLY-78	77.63 ± 0.24	68.17 ± 0.30	65.20 ± 0.40	4.99 ± 0.02	1.86 ± 0.01	2.70 ± 0.02	9.33 ± 0.41	1.94 ± 0.07	5.0	23.88 ± 0.20
GLY-105	77.47 ± 0.23	66.20 ± 0.44	61.23 ± 0.35	5.00 ± 0.04	1.81 ± 0.01	2.75 ± 0.02	8.67 ± 0.26	1.77 ± 0.04	5.5	24.30 ± 0.13
GLY-123	77.40 ± 0.31	67.87 ± 0.20	63.43 ± 0.29	5.08 ± 0.02	1.83 ± 0.02	2.72 ± 0.02	8.67 ± 0.12	1.71 ± 0.02	5.0	23.91 ± 0.14
GLY-147	76.47 ± 0.20	67.73 ± 0.18	64.40 ± 0.29	5.11 ± 0.04	1.83 ± 0.02	2.71 ± 0.01	8.63 ± 0.15	1.77 ± 0.02	5.0	24.53 ± 0.09
GLY-163	76.80 ± 0.21	66.87 ± 0.20	64.87 ± 0.20	4.97 ± 0.06	1.79 ± 0.03	2.72 ± 0.02	8.60 ± 0.06	1.69 ± 0.03	5.5	24.14 ± 0.07
GLY-169	77.43 ± 0.15	67.77 ± 0.32	63.37 ± 0.30	4.83 ± 0.04	1.74 ± 0.02	2.75 ± 0.01	8.13 ± 0.18	1.69 ± 0.03	5.0	23.06 ± 0.25
GLY-178	77.27 ± 0.15	67.80 ± 0.38	61.63 ± 0.54	4.80 ± 0.04	1.78 ± 0.01	2.69 ± 0.03	8.60 ± 0.12	1.79 ± 0.02	5.0	24.55 ± 0.14
GLY-196	77.37 ± 0.18	69.00 ± 0.25	66.80 ± 0.44	5.03 ± 0.02	1.73 ± 0.02	2.83 ± 0.02	8.63 ± 0.18	1.70 ± 0.02	5.5	23.88 ± 0.42
GLY-209	76.70 ± 0.17	65.67 ± 0.47	61.03 ± 0.90	4.90 ± 0.02	1.76 ± 0.02	2.75 ± 0.02	8.90 ± 0.32	1.85 ± 0.07	5.5	20.58 ± 0.63
GLY-221	77.10 ± 0.15	68.30 ± 0.38	64.03 ± 0.46	4.85 ± 0.08	1.74 ± 0.03	2.71 ± 0.03	8.50 ± 0.23	1.77 ± 0.06	5.0	23.24 ± 0.50
GLY-247	77.67 ± 0.18	68.83 ± 0.38	64.43 ± 0.29	4.86 ± 0.03	1.83 ± 0.02	2.69 ± 0.02	8.37 ± 0.15	1.67 ± 0.03	6.0	23.89 ± 0.41
GLY-286	76.93 ± 0.18	66.77 ± 0.49	60.57 ± 0.58	4.85 ± 0.05	1.79 ± 0.02	2.72 ± 0.02	9.17 ± 0.29	1.94 ± 0.05	5.5	24.50 ± 0.37
GLY-302	77.43 ± 0.24	67.40 ± 0.32	63.50 ± 0.44	4.97 ± 0.03	1.75 ± 0.01	2.81 ± 0.05	9.57 ± 0.46	1.95 ± 0.07	5.5	24.44 ± 0.25
GLY-327	76.93 ± 0.15	66.73 ± 0.44	60.50 ± 0.52	4.80 ± 0.04	1.79 ± 0.02	2.66 ± 0.03	8.73 ± 0.41	1.85 ± 0.07	5.5	24.58 ± 0.23
GLY-339	76.80 ± 0.32	67.10 ± 0.61	62.40 ± 0.44	4.84 ± 0.02	1.81 ± 0.02	2.75 ± 0.02	9.53 ± 0.55	1.97 ± 0.10	5.0	23.25 ± 0.35
ISM	77.27 ± 0.12	67.83 ± 0.20	64.33 ± 0.43	5.01 ± 0.03	1.80 ± 0.02	2.73 ± 0.01	8.67 ± 0.15	1.77 ± 0.02	5.0	24.39 ± 0.15
PR 114 (Xa38)	78.20 ± 0.17	66.70 ± 0.29	65.20 ± 0.40	5.98 ± 0.08	2.21 ± 0.02	2.69 ± 0.02	11.40 ± 0.26	2.23 ± 0.03	6.0	23.80 ± 0.18
CV (%)	0.48	0.73	0.72	1.36	1.83	1.63	4.91	3.10		1.79
LSD (*P = 0*.*05*)	0.61	0.80	0.75	0.11	0.05	0.07	0.72	0.09		0.69

Coefficient of variance (CV); Least significant difference (LSD) at 5% probability level; HRR, Head rice recovery; KL, Kernel length; KB, Kernel breadth; L/B ratio, Length/Breadth; KLAC, Kernel length after cooking; ER, Elongation ratio; ASV, Alkali spreading value; AC, Amylose content.

## Discussion

BB is a major production problem in northwestern, eastern and parts of northeastern India, particularly the states of Assam and Tripura, and on the entire eastern and southern coast of India including parts of Kerala, Karnataka and Konkan regions of Maharashtra [4]. Rice disease survey data indicate that BB has increased significantly in both intensity and geographical spread based on reports of BB occurrence in epidemic form in several rice growing regions in India [4, 43]. Development and deployment of resistant rice varieties offer the best alternative approach to disease management. More than 40 BB-resistant genes were characterized from diverse sources [12, 13]. However, several studies showed that a single BB-resistant gene cannot provide broad-spectrum resistance to BB in India [5, 16–17] because of several races of the pathogen in a particular rice-growing region. Thus, a continuous effort must increase the durability of resistance either by stacking additional resistance genes in existing varieties or by diversification of resistance genes through identification of new ones [25].

Pyramiding of multiple BB-resistant genes can be achieved using marker-assisted selection (MAS) in a backcross breeding method. In India, MAS was successfully used to develop BB resistant rice varieties like Improved Pusa Basmati 1 (*Xa21* + *xa13*) and ISM (*Xa21* + *xa13* + *xa5*) [20, 27]. Although newly released varieties such as ISM currently provide a good level of resistance to BB under field conditions in most of the rice growing regions in India, the durability of resistance is threatened by the appearance of new and more virulent races of the pathogen. In fact, strains in the Indian *Xoo* population are reported that can cause moderate susceptibility on IRBB55, which possesses a combination of *Xa21* and *xa13* [5, 16, 44]. Incidentally, ISM has shown slightly larger lesions with a disease score of 5 upon artificial inoculation at certain BB hot spot locations (Kaul and Pantnagar) in different All India Coordinated Plant Pathology trials [30]. These observations indicate that novel resistance genes must be identified and deployed in the genetic background of elite Indian rice varieties to develop broad-based resistance.

ISM has become a highly popular rice variety occupying more than 80,000 ha among farmers in southern India because of the highly preferred grain quality, which is equivalent to Samba Mahsuri, and the in-built resistance to BB. To increase the spectrum and durability of resistance of ISM against BB, we added a newly identified, broad-spectrum BB-resistant gene, *Xa38*, into ISM through MABB in the present study. The BB-resistant gene *Xa38* was identified from an accession of *Oryza nivara* (Acc No. IRGC 81825) [31] and was mapped in the long arm of chromosome 4. The gene was further fine-mapped to a genomic region of 38.4 kb, and a highly reliable InDel marker (Os04g53050-1) was developed based on the putative candidate gene [10, 14]. The gene shows a broad-spectrum resistance to races from Punjab Province and *Xoo* strains from other rice growing regions [14, 31, 44]. Additionally, in the present study, the breeding line containing *Xa38*, viz., PR 114 (Xa38), showed a high level of resistance to most of the strains of *Xoo*, which indicated that this gene was indeed the correct choice for increasing the resistance of ISM against BB disease.

MABB is the most commonly used strategy for introgressing one or a few target genes into crop varieties [45], and with this strategy, varieties have been improved for desired traits [12]. Precise foreground selection is the key to the success of MABB [46], and the highly reliable marker Os04g53050-1 was used for detection of *Xa38* [10]. For detection of *Xa21*, *xa13* and *xa5*, gene-linked markers pTA248 [33], xa13-Prom [25, 34] and 10603-T10Dw [35], respectively, were used. Foreground selection of the target BB-resistant genes was coupled with background selection using a set of polymorphic SSR markers (# 83) to identify the plants (BCF_1_s) with the maximum RPG recovery percentage at each backcrossing step. The number of polymorphic SSRs was 18 on target chromosome 4 and ~5–7 per chromosome for non-target chromosomes. The use of polymorphic SSRs to identify the plants with maximum RPG recovery percentage helped to reduce the number of backcrosses, and this approach has been used in several other studies [14, 20, 27, 47].

The true F_1_s (F_1_, BC_1_F_1_, BC_2_F_1_ and BC_3_F_1_) were identified with the *Xa38* gene-specific marker Os04g53050-1, and the positive plants were then further screened with parental polymorphic markers to identify the plants with the maximum RPG recovery. The extent of RPG in BC_3_F_1_s was 92.7–95.1%, which was possible because in the present study, we conducted background selection with a high number of parental polymorphic SSR markers (# 83) covering the entire genome at each backcross generation, particularly focusing on the target chromosome (i.e., Chr. 4 on which *Xa38* is located), coupled with stringent phenotypic selection. Earlier workers also reported accelerated recovery of the RPG within two or three backcross generations [14, 27, 38, 47]. A selected plant from the BC_3_F_1_ stage with maximum RPG, phenotypic similarity with the recurrent parent and very high level of BB resistance was selfed to generate BC_3_F_2_. In addition to BB resistance, stringent selection was conducted to identify any plants with agro-morphological characters and yield better than those of the recurrent parent. A total of 18 entries from BC_3_F_2_ were selected that possessed four BB-resistant genes (*Xa38*, *Xa21*, *xa13* and *xa5*) in the homozygous condition and had agro-morphological characters and yield superiority over the recurrent parent. The introgression of the donor parent chromosomal segment in most of these 18 selected entries was restricted on either side of the target gene to a small region of ~0.6 Mb of *Xa38* (Fig 3). These lines had RPG recovery in the range of 92.7–95.1%. These entries were advanced to the BC_3_F_6_ generation for analysis of grain quality and agro-morphological characters.

All the selected 4-gene pyramided improved lines showed a very high level of BB resistance against all eight *Xoo* strains used in the present study. The average lesion length among the BDLs was less than 0.5 cm, whereas the recurrent parent ISM showed a lesion length ranging from 2.07 to 6.43 cm (Table 1). The data indicated that the addition of *Xa38* in the genetic background of ISM (in addition to *Xa21*, *xa13* and *xa5*) increased the level of resistance against BB. Analysis of *Xoo* populations from India shows strains individually virulent on *Xa21*, *xa13* and *xa5* [5, 16–17, 44]. All four genes selected for the present study have different modes of action against *Xoo* [20, 31], and when presented together, these genes imparted a good level of resistance to BB. Dominant BB resistant gene, *Xa21* encodes a receptor kinase containing NBS-LRR domain while *xa5* encodes a mutated form of basal transcription factor, TFIIAγ5 [5, 20]. Recessive BB resistant gene, *xa13* has a mutation in the promoter of *Os8N3/OsSWEET11*, a host susceptibility gene that encodes a sugar transporter [48]. BB resistant gene *Xa38*, identified from *O*. *nivara* seems to be of NBS-LRR type based on the putative candidate gene sequence analysis [10]. We understand that due to the very dynamic nature of the pathogen, the durability of resistance is threatened due to continuous evolution of virulence. The addition of a broad-spectrum BB-resistant gene, *Xa38*, to the existing gene combination of *Xa21 + xa13 + xa5* in ISM is expected to increase the durability of resistance of this precious rice variety.

The retention or improvement of different agro-morphological traits, the grain quality and the yield of the original recurrent parent in the backcross-derived gene pyramid lines is crucial in any MAS program. In addition to the marker-assisted introgression of specific traits, MABB programs also focus on stringent phenotypic selection for retaining or further improvement of agro-morphological characters and yield in the advanced lines in comparison with the original recurrent parent [14, 20, 27, 38, 47]. When the selected 18 advanced lines were evaluated for different agro-morphological traits and yield, many lines showed superiority over the recurrent parent, particularly for plant height, days to 50% flowering, number of grains/panicle and grain weight/panicle (Table 2). In the present study, in addition to the increased level of BB resistance, with stringent selection, we could identify plants with yield and other agro-morphological characters equivalent or superior to the recurrent parent. ISM has a medium-slender, fine-grain type compared with the long-slender grain type of the donor parent (which is not preferred). However, with strong phenotype selection based on grain characters and plant morphology at the BC_3_F_2_ stage, we could select the few plants that were agronomically superior to ISM but also possessed the desirable grain quality of ISM. All the backcross-derived improved lines of ISM were equivalent to ISM for most of the grain quality characters.

In conclusion, in the present study, we developed improved versions of ISM by the addition of the novel, broad-spectrum BB-resistant gene *Xa38*, and these lines exhibited high levels of BB resistance to multiple *Xoo* isolates. Furthermore, many of these improved lines showed superior agro-morphological characters and yield to those of the recurrent parent, whereas most of the desirable grain and quality characters were retained. Selected lines are nominated in multi-location trials under the All India Coordinated Rice Improvement Project (AICRIP) for their possible release for the benefit of rice farmers.
